# Prediction of hepatocellular carcinoma response to ^90^Yttrium radioembolization using volumetric ADC histogram quantification: preliminary results

**DOI:** 10.1186/s40644-019-0216-6

**Published:** 2019-05-29

**Authors:** Sonja Gordic, Mathilde Wagner, Riccardo Zanato, Stefanie Hectors, Cecilia Besa, Shingo Kihira, Edward Kim, Bachir Taouli

**Affiliations:** 1 Translational and Molecular Imaging Institute, Icahn School of Medicine at Mount Sinai, New York, NY USA; 2 Institute of Diagnostic and Interventional Radiology, University Hospital Zurich, Zurich, Switzerland; 3 Sorbonne Universités, UPMC, Department of Radiology, Hôpital Pitié-Salpêtrière, Assistance Publique-Hôpitaux de Paris, Paris, France; 4 Department of Radiology, San Bassiano Hospital, Bassano del Grappa, Vicenza, Italy; 5 Department of Radiology, School of Medicine, Pontificia Universidad Católica de Chile, Avenida Libertador Bernardo O’Higgins 340, 8331150 Santiago, Chile; 6 Department of Radiology, Icahn School of Medicine at Mount Sinai, One Gustave L. Levy Place, Box 1234, New York, NY 10029-6574 USA

**Keywords:** Hepatocellular carcinoma, Radioembolization, Diffusion-weighted MRI

## Abstract

**Purpose:**

To assess the predictive value of volumetric apparent diffusion coefficient (vADC) histogram quantification obtained before and 6 weeks (6w) post-treatment for assessment of hepatocellular carcinoma (HCC) response to ^90^Yttrium radioembolization (RE).

**Methods:**

In this retrospective study, 22 patients (M/F 15/7, mean age 65y) who underwent lobar RE were included between October 2013 and November 2014. All patients underwent routine liver MRI pre-treatment and 6w after RE. Two readers assessed index tumor response at 6 months after RE in consensus, using mRECIST criteria. vADC histogram parameters of index tumors at baseline and 6w, and changes in vADC (ΔvADC) histogram parameters were calculated. The predictive value of ADC metrics was assessed by logistic regression with stepwise parameter selection and ROC analyses.

**Results:**

Twenty two HCC lesions (mean size 3.9 ± 2.9 cm, range 1.2–12.3 cm) were assessed. Response at 6 months was as follows: complete response (CR, *n* = 6), partial response (PR, *n* = 3), stable disease (SD, *n* = 12) and progression (PD, n = 1). vADC median/mode at 6w (1.81–1.82 vs. 1.29–1.35 × 10^− 3^ mm^2^/s) and ΔvADC median/max (27–44% vs. 0–10%) were significantly higher in CR/PR vs. SD/PD (*p* = 0.011–0.036), while there was no significant difference at baseline. Logistic regression identified vADC median at 6w as an independent predictor of response (CR/PR) with odds ratio (OR) of 3.304 (95% CI: 1.099–9.928, *p* = 0.033) and AUC of 0.77. ΔvADC mean was identified as an independent predictor of CR with OR of 4.153 (95%CI: 1.229–14.031, *p* = 0.022) and AUC of 0.91.

**Conclusion:**

Diffusion histogram parameters obtained at 6w and early changes in ADC from baseline are predictive of subsequent response of HCCs treated with RE, while pre-treatment vADC histogram parameters are not. These results need confirmation in a larger study.

**Trial registration:**

This retrospective study was IRB-approved and the requirement for informed consent was waived.

## Background

Hepatocellular carcinoma (HCC) is the 6th most common malignancy and the second most common cause of cancer-related mortality worldwide [1]. Radical treatment options including liver transplantation and surgical resection are available only in a small number of cases with HCC. Liver transplantation is usually performed in patients with cirrhosis and HCC within the Milan criteria [2]. Owing to limited availability of donor organs the waiting time prolongs and thus increases the chance of dropout due to tumor progression [3]. ^90^Yttrium radioembolization (RE) has been demonstrated to be safe and effective [4, 5] and can be used for downstaging or bridging of patients listed for liver transplantation [6–8]. Therefore, the evaluation of tumor response after RE is essential in directing clinical management, for indication of repeat treatment and prognostication.

The modified Response Evaluation Criteria in Solid Tumors (mRECIST), using the single largest diameter of the arterially hyperenhancing viable tumor, is currently proposed as the standard methodology to assess radiological response in HCC [9]. mRECIST is primarily based on contrast-enhanced computed tomography (CT) or T1-weighted imaging (CE-T1WI) with magnetic resonance imaging (MRI). Although mRECIST has been shown to predict survival post transarterial chemoembolization (TACE) [10–14], a unified consensus on an early imaging biomarker to assess HCC tumor response and outcome post RE has not been reached.

Diffusion-weighted imaging (DWI) provides information on cell membrane integrity and cellular density. Several studies have suggested that apparent diffusion coefficient (ADC) is an earlier surrogate of response compared to size criteria in HCC treated by RE [15–18]. Kamel et al. [19–24] first developed the use of volumetric ADC (vADC) in this setting. A survival benefit was demonstrated in cholangiocarcinoma and neuroendocrine liver metastases treated by TACE exhibiting response by vADC [19, 23, 24]. One of these studies focused on the identification of volumetric functional response criteria of HCC treated by TACE and confirmed that vADC potentially enables patient stratification for survival [19, 21]. To the best of our knowledge, there is only one study exploring the use of vADC in HCC post RE, in which the authors concluded that vADC performed better than RECIST in detecting response using liver explant as the reference, without being able to predict complete pathological necrosis [25].

First order radiomics features (histogram analysis) assess the spectrum of ADC values obtained from all voxels within a volume of interest. The information about the distribution of ADC values within the tumor can offer valuable additional insights into tumor structure and heterogeneity [26–33]. This information could be of benefit in predicting response and prognosis in HCC post RE.

The aim of our preliminary study was to assess the predictive value of histogram quantification measured on vADC obtained before and at 6 weeks (6w) post-treatment for assessing HCC response to RE as assessed by mRECIST criteria at 6 months (6 m).

## Material and methods

### Patients

This retrospective study was IRB-approved and the requirement for informed consent was waived. Our institutional database was queried between October 2013 and November 2014, to identify patients who had HCC and underwent MRI before and after RE. Fifty five patients were identified. Thirty three patients were excluded for the following reasons: previous RE (*n* = 12), previous TACE (n = 1), no follow-up imaging/CT follow-up at 6w or 6 m (*n* = 9), and different b-values for the DWI acquisitions at baseline and 6w (*n* = 11). The final study group comprised 22 patients (M/F 15/7, mean age 65y). Diagnosis of HCC was based on OPTN criteria [34] (*n* = 17) or tissue sampling (*n* = 5). Five patients had a histologically proven moderately differentiated HCC. Fifteen patients underwent lobar RE of the right lobe and 7 patients of the left lobe. The exclusive lobar RE treatment reflects our initial experience with RE, which now has evolved to more segmental treatments. All patients had cirrhosis, with the following etiologies: chronic hepatitis C (*n* = 14), chronic hepatitis B (*n* = 3), nonalcoholic steatohepatitis (n = 3), alcohol abuse (*n* = 1) or cryptogenic cirrhosis (n = 1). The median alpha-fetoprotein (AFP) before RE was 22.9 and after RE 10 ng/ml. Patient’s characteristics are given in Table 1.

**Table 1:** Population characteristics

Variable	N	%
Age	65 ± 6	
Sex (M/F)	15/7	68/32
Etiology of liver disease		
Chronic HCV	14	64
Chronic HBV	3	14
NASH	3	14
Alcohol abuse	1	4
Cryptogenic	1	4
Alpha-fetoprotein		
Pre-treatment [median (range), ng/ml]	22.95 (0.9–11.309)	
Post-treatment [median (range), ng/ml]	10 (2.4–31.813)	
Child-Pugh Score A/B/C	19/3/0	86/14/0
Radiation dose [mean ± SD, GBq]	2.1 ± 1.5	
Time between pre-treatment MRI and RE [mean ± SD, days]	60 ± 22	
Time between radioembolization and 6w follow-up imaging [mean ± SD, days]	57 ± 21	
Time between RE and follow-up MRI [mean ± SD, days]	137 ± 41	

### MRI acquisition

Imaging was performed with different clinical systems (baseline, 6w and 6 m): 3 T GE 750 (*n* = 10), 1.5 T GE Signa (*n* = 17), 3 T Siemens Skyra (*n* = 11) or 1.5 T Siemens Aera (*n* = 28). Routine liver MRI protocol included non-fat suppressed axial and coronal single-shot fast spin-echo T2-weighted imaging (WI) (HASTE/SSFSE), axial fat suppressed fast spin echo (FSE) T2WI, T1WI in- and out-of- phase, diffusion-weighted imaging and dynamic contrast-enhanced (CE)-T1WI including image subtraction.

For dynamic CE-T1WI, unenhanced, early (AP1) and late arterial phases (AP2), portal venous phase (PVP) (60s), transitional phase (TP) (180 s), and hepatobiliary phase (HBP) (at 10 and 20 min) were obtained using a 3D T1WI breath-hold fat-suppressed spoiled gradient-recall echo sequence (VIBE or LAVA) before and after administration of gadoxetic acid disodium (Primovist/Eovist, Bayer HealthCare). A fixed dose of 10 ml of contrast (mean weight-based dose of 0.03 mmol/kg) was injected at a rate of 1.5 ml/s followed by a 20 ml saline flush using a bolus tracking method.

DWI was performed in the axial plane with tri-directional diffusion gradients using 3 b-values (50, 400, and 800 s/mm^2^). DWI was acquired after contrast administration. Previous studies have shown that there is no significant difference in the ADC values of focal hepatic lesions before and after administration of gadolinium contrast [35, 36]. ADC maps using a monoexponential diffusion model with the 3 b-values were automatically generated from the MRI systems. Acceptable interplatform reproducibility in ADC values has been reported, including between 1.5 T and 3 T systems [37–41]. Recently, we have reported excellent inter-platform reproducibility (1.5 T vs 3 T) of ADC using a dedicated DWI phantom (coefficient of variation CV < 7%), as well as in healthy volunteers (CV < 13.5%) [39].

### Image analysis

#### Lesion selection

The study coordinator (MW, with 5 years of experience in abdominal MRI) reviewed clinical data, and images using a PACS, and identified the largest index tumor that underwent RE. If multiple lesions were present in the same lobe, only the largest HCC (≥ 1 cm) was assessed. Size, segment location, series number(s) and image number(s) where the lesion was visualized on the 6 m follow-up imaging and was recorded for each HCC to ensure that the readers analyzing the data assessed the same lesions. Anonymized evaluation sheets, providing this information, were given to the two observers who analyzed the images qualitatively and quantitatively.

#### Qualitative image analysis

Two observers (SG, and CB, with 2 and 5 years of experience in abdominal MRI, respectively) reviewed the images at baseline, 6w and 6 m in consensus in random order, and assessed mRECIST (in cm) on native, contrast-enhanced and subtracted T1W images at 6 m follow-up MRI on the index lesions. Both observers were informed of the presence and location of HCCs, but they were blinded to clinical data and ADC values.

#### Quantitative image analysis

All series, including ADC maps, of each baseline and 6w follow-up MRI scans were transferred to a workstation equipped with a dedicated software (OsiriX, Bernex, Switzerland) permitting volumetric tumor delineation and data analysis. One observer (SG, with 2 years of experience in abdominal MRI) performed the quantitative analysis 6w after the qualitative image analysis to decrease recall bias. Freehand regions of interest (ROIs) were placed on the whole index tumors, including necrotic portions. Volumes of interest (VOIs) were acquired by drawing ROIs on each slice of the ADC map where the tumor was delineated. Tumor size was measured on the axial post-contrast T1-weighted arterial phase or portal venous images. Volumetric tumor delineation on ADC was aided by registering to other DWI and CE-T1WI, and by delineating areas suspicious of tumor (diffusion restriction, arterial enhancement). All scanner generated ADC maps and ROIs at baseline and 6w follow-up of each patient were exported and tumor volume and vADC histogram parameters (mean, median, mode, min, max, kurtosis and skewness) were calculated subsequently using a MATLAB script (The Mathworks, Inc., Natick, MA).

$$ \Delta \mathrm{vADC}\ \mathrm{was}\ \mathrm{measured}\ \mathrm{as}:\Delta \mathrm{vADC}\ \left(\%\right)=\left[\left(\mathrm{vADC}\ 6\mathrm{w}-\mathrm{vADC}\ \mathrm{baseline}\right)/\mathrm{vADC}\ \mathrm{baseline}\right]\times \kern0.37em 100. $$

#### Reference standard

The reference standard was defined as response of the target lesion at 6 months as assessed by mRECIST [9]: 1) complete response (CR): disappearance of intratumoral arterial hyperenhancement in target lesion; 2) partial response (PR): ≥ 30% decrease in the sum of diameter of viable (intratumoral arterial hyperenhancement) target lesion; 3) stable disease (SD): no qualification for PR or PD; 4) progressive disease (PD): ≥ 20% increase in the sum of diameters of viable (intratumoral arterial hyperenhancement) target lesion.

#### Statistical analysis

Quantitative variables were expressed as mean ± standard deviation and categorical variables as frequencies or percentages. A Mann-Whitney U test was used to test for significant differences between AFP, vADC mean, median, mode, min, max, kurtosis and skewness at baseline and 6w. A multivariable logistic regression analysis with stepwise parameter selection using Wald tests was used to test imaging variables (baseline ADC, ADC 6w and ΔvADC), lesion size (diameter and volume) and AFP values as predictors of any tumor response (partial response/ complete response) and complete response in the index lesion at 6 months. Prior to regression analysis, parameters were standardized to have zero mean and unit standard deviation. Receiver operating characteristics (ROC) analysis was performed for the parameters selected by the logistic regression procedure to assess the utility of the measures for the detection of response at 6 months. All statistical analyses were conducted using SPSS software (release 21.0; SPSS, Chicago, Il). A two-tailed *p*-value less than 0.05 was considered to indicate a significant difference.

## Results

Twenty-two HCC lesions with a mean diameter of 3.9 ± 2.9 cm (range 1.2–12.3 cm) were assessed in 22 patients. Fifteen lesions were located in the right hepatic lobe and 7 in the left hepatic lobe. The response at 6 m was as follows: 6 tumors with complete response (CR, 27.2%), 3 tumors with partial response (PR, 13.6%), 12 tumors with stable disease (SD, 54.6%) and 1 tumor with progression (PD, 4.6%).

### Quantitative analysis

**Fig. 1:**
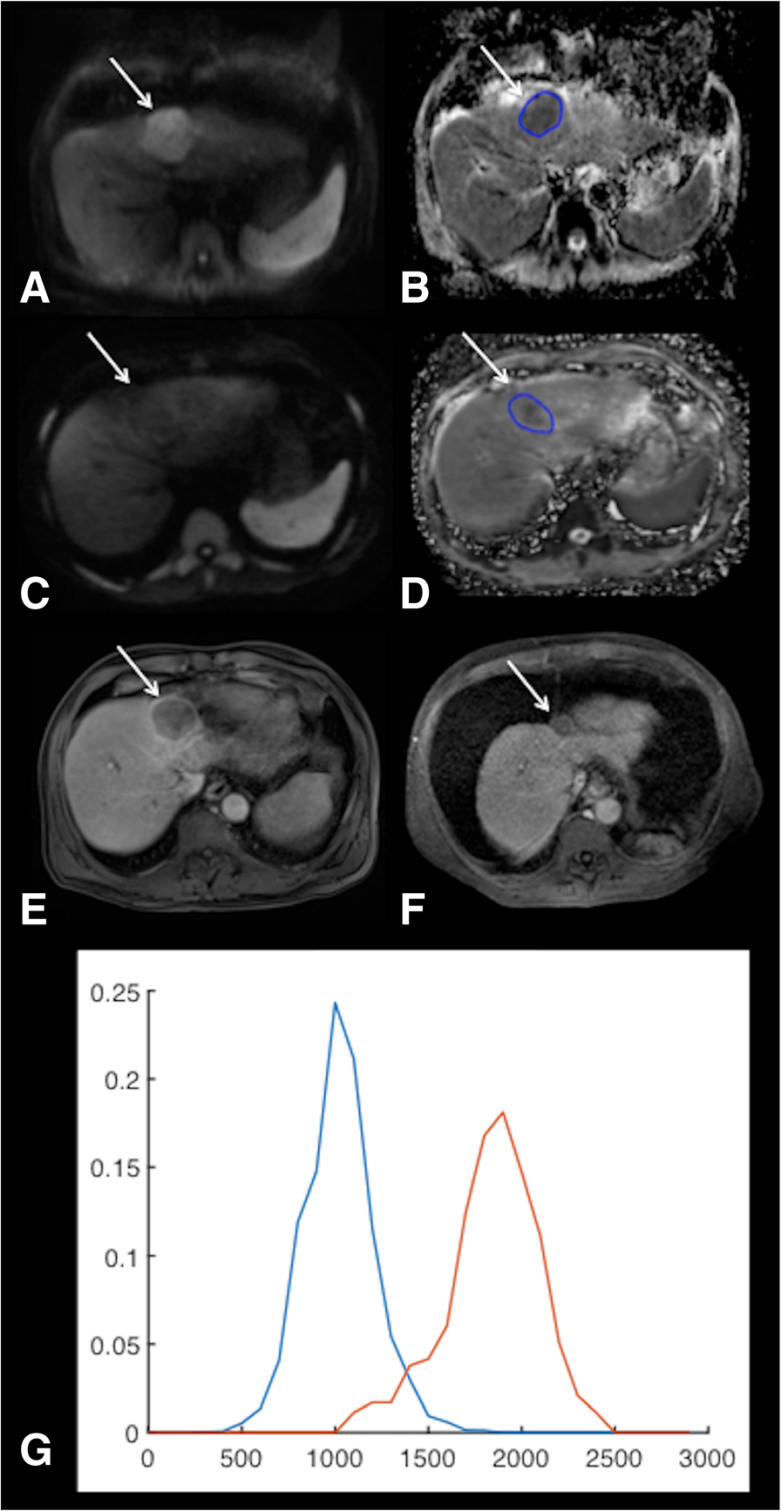
63-year-old male patient with HCV cirrhosis and HCC treated with radioembolization (RE). Pre-treatment MRI: HCC (arrows) demonstrates restricted diffusion with high signal on high b value DWI (b 800) (**a**) and low ADC on ADC map (**b**). Post-treatment MRI at 6w post RE shows resolution of diffusion restriction on DWI (b 800) (**c**) with residual low ADC areas in tumor (**d**). Contrast-enhanced-T1weighted images obtained during the portal-venous phase at baseline (**e**) and 6 months (**f**) show complete tumor response at 6 months. Corresponding histogram distribution of vADC at baseline (blue, vADC median 1.08 × 10^− 3^ mm^2^/s, vADC mean 1.07 × 10^− 3^ mm^2^/s) and 6w after RE (red, vADC median 1.91 × 10^− 3^ mm^2^/s, vADC mean 1.89 × 10^− 3^ mm^2^/s, ΔvADC median 78%, ΔvADC mean 76%) shows a shift of the distribution to the right after RE (**g**)

Pre- and post-treatment serum AFP levels were not significantly different in patients with PR/CR vs. those with SD/PD (*p* = 0.456 and *p* = 0.554, respectively). No significant difference was observed in tumor size and volume in patients with PR/CR and SD/PD at baseline and at 6w (Table 2). There was no significant difference in pre-treatment vADC parameters between patients with PR/CR vs. those with SD/PD, while vADC median and mode were significantly higher at 6w in patients with PR/CR vs. those with SD/PD. Furthermore, ΔvADC median and ΔvADC max were found to be significantly higher in patients with PR/CR vs. those with SD/PD **(**Table 2, Fig. 1).

**Table 2:** Volumetric ADC histogram measurements (vADC) obtained at baseline and 6 weeks (6w) post ^90^Yttrium radioembolization in index tumors, as well as differences between baseline and 6w

	Baseline			6w			Δ (%)		
	PR/CR	SD/PD	p	PR/CR	SD/PD	p	PR/CR	SD/PD	p
Maximum diameter (cm)	3.02 ± 1.08	4.55 ± 3.61	0.65	4.11 ± 2.56	4.25 ± 3.43	0.56	61.7 ± 172.9	−4.1 ± 12.1	0.47
Volume (cm^3^)	19.75 ± 29.78	176.29 ± 345.45	0.33	12.84 ± 22.02	149.67 ± 308.98	0.27	153.3 ± 286.2	140.7 ± 308.7	0.54
vADC mean (10^− 3^ mm^2^/s)	1.34 ± 0.49	1.27 ± 0.22	0.85	1.82 ± 0.48	1.36 ± 0.26	0.06	43.3 ± 40.1	8.3 ± 21.5	0.05
vADC median (10^−3^ mm^2^/s)	1.35 ± 0.52	1.25 ± 0.22	1	1.82 ± 0.47	1.35 ± 0.25	**0.04**	43.6 ± 41.2	10.3 ± 20.9	**0.04**
vADC mode (10^−3^ mm^2^/s)	1.36 ± 0.66	1.06 ± 0.39	0.21	1.81 ± 0.45	1.29 ± 0.30	**0.02**	46.1 ± 45.2	108.1 ± 336.7	0.19
vADC min (10^−3^ mm^2^/s)	0.67 ± 0.31	0.67 ± 0.36	0.39	1.21 ± 0.57	0.83 ± 0.44	0.13	118.2 ± 166.2	80.9 ± 240.6	0.24
vADC max (10^−3^ mm^2^/s)	2.00 ± 0.56	2.32 ± 0.85	0.43	2.46 ± 0.55	2.25 ± 0.80	0.51	27.2 ± 32.2	−0.0 ± 27.8	**0.01**
vADC kurtosis	3.32 ± 1.13	4.53 ± 3.00	0.26	3.18 ± 0.57	4.05 ± 1.73	0.43	8.5 ± 47.1	−2.3 ± 33.6	0.79
vADC skewness	0.38 ± 0.48	0.66 ± 0.57	0.24	−0.03 ± 0.53	0.35 ± 0.54	0.16	− 141.3 ± 120.2	−125.6 ± 142.1	0.56

*PR* partial response, *CR* complete response, *SD* stable disease, *PD* progressive disease

Significant *p*-values are bolded

**Fig. 2:**
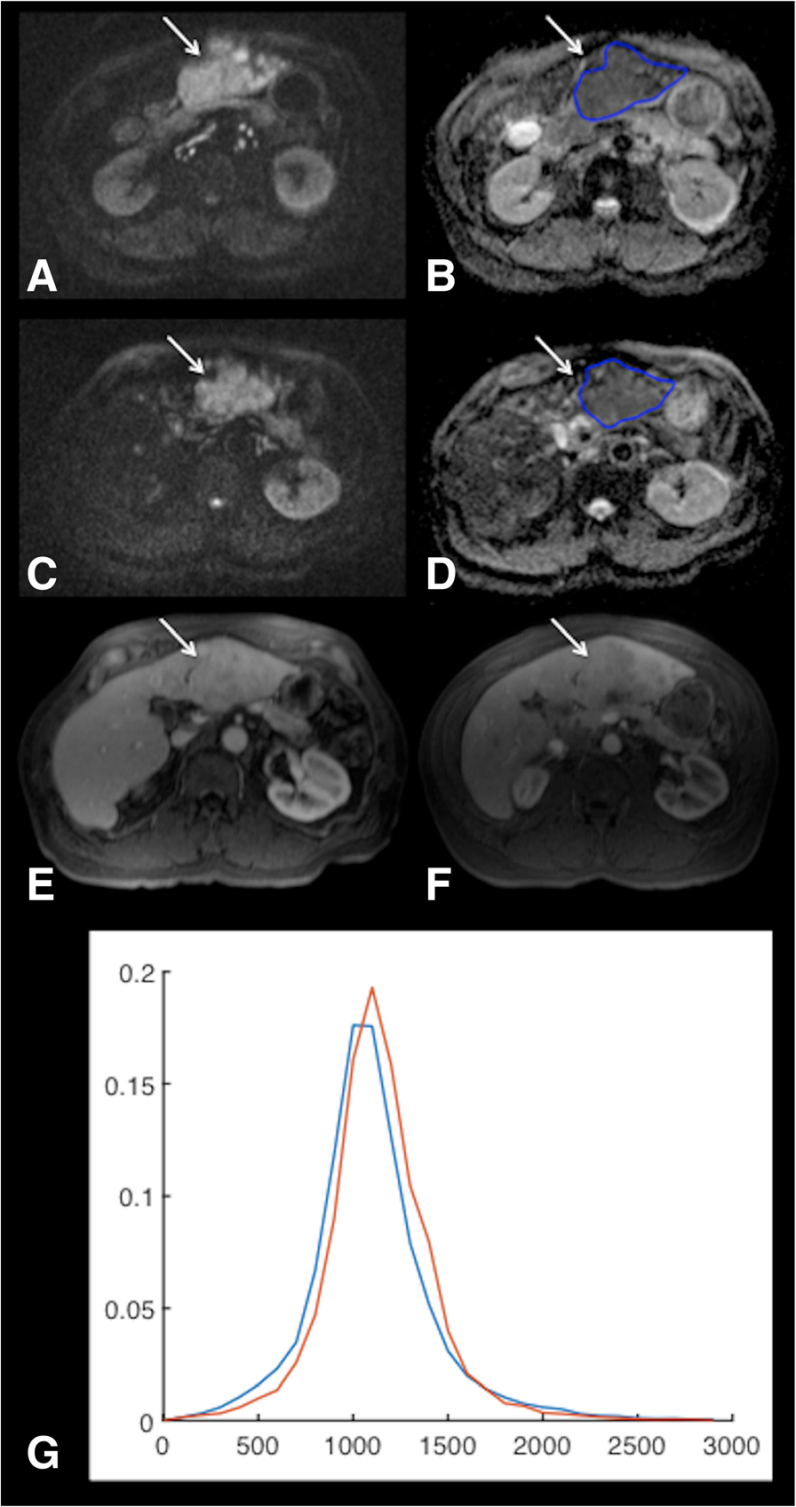
69-year-old male patient with HCV cirrhosis and HCC treated with radioembolization (RE). Pre-treatment MRI: HCC (arrows) demonstrates restricted diffusion on high b value DWI (b 800) (**a**) and ADC map (**b**). Post-treatment MRI at 6w post RE shows unchanged diffusion restriction on DWI (b 800) (**c**) with low ADC (**d**). Contrast-enhanced-T1 weighted images obtained during the portal-venous phase at baseline (**e**) and at 6 months (**f**) show stable disease at 6 months. Corresponding histogram distribution of vADC before (blue, vADC median 1.12 × 10^− 3^ mm^2^/s, vADC mean 1.12 × 10^− 3^ mm^2^/s) and 6w after RE (red, vADC median 1.17 × 10^− 3^ mm^2^/s, vADC mean 1.19 × 10^− 3^ mm^2^/s, ΔvADC median 4%, ΔvADC mean 3%) shows an almost identical distribution after RE (**g**)

Using a multivariable logistic regression analysis with stepwise parameter selection, we identified vADC median at 6w as an independent predictor of any response [PR/CR; odds ratio (OR) 3.304, *p* = 0.033)], while ΔvADC mean was identified as an independent predictor of CR (OR 4.153, *p* = 0.022). A vADC median threshold of 1.901 × 10^− 3^ mm^2^/s at 6w had a sensitivity of 67% and a specificity of 100% for prediction of PR/CR, while a ΔvADC mean threshold of 19.2% had a sensitivity of 100% and a specificity of 81% for prediction of CR **(**Table 3, Figs. 1, 2).

**Table 3:** Logistic regression with stepwise selection of parameters for prediction of response (PR/CR and CR)

Parameter	Logistic regression		ROC analysis				
	OR (CI95%)	p	AUC	p	Threshold	Sensitivity (%)	Specificity (%)
PR/CR							
vADC median 6w (10^−3^ mm^2^/s)	3.304 (1.099–9.928)	0.033	0.77 (0.53–1.00)	0.035	1.901	66.7	100.0
CR							
ΔvADC mean (%)	4.153 (1.229–14.031)	0.022	0.91 (0.78–1.00)	0.004	19.2	100.0	81.3

*AUC* area under the curve, *CR* complete response, *OR* odds ratio, *PR* partial response

Results of logistic regression and ROC analysis of the parameters selected by the logistic regression procedure are shown

## Discussion

In this preliminary study, we have evaluated the potential of vADC histogram measurements obtained pre- and early (6w) post-treatment for prediction of HCC response to RE. Volumetric histogram parameters vADC median, mode, ΔvADC median and ΔvADC max obtained at 6w post-treatment were significantly different between patients with PR/CR vs. those with SD/PD. Furthermore, ΔvADC mean was an independent predictor of complete response at 6 m, while vADC median was an independent predictor of any response (partial and complete) at 6 m. Pre-treatment vADC did not have any predictive value for response at 6 m.

mRECIST is currently proposed as the standard method to assess radiological response of HCC [9] and has been shown to predict survival in patients with HCC post TACE [10–13]. However, a unified consensus on an early imaging biomarker to assess HCC tumor response and outcome post RE has not been reached.

Two MRI-pathological correlation studies [42, 43] from the same group of investigators showed potential of the World Health Organization (WHO) and the European Association for the Study of the Liver (EASL) criteria to predict complete pathological necrosis in patients with HCC post-RE. Such correlation was not observed in patients with HCC post RE ± sorafenib in a recent study [44] by the same group.

DWI provides information on cell membrane integrity and cellular density. Several studies have suggested that ADC can be used as earlier surrogate of response compared to size criteria in HCC treated by RE [15–17]. Kokabi et al. [45] reported that ADC measured at 30 days in 18 patients post RE was able to predict HCC response at 3 months with a sensitivity of 90%. A > 30% increase in ADC value at 30 days, furthermore, predicted significantly prolonged survival. Also, Niekamp et al. [46] showed that a pre-procedure ADC < 1.01 × 10^− 3^ mm^2^/s is an independent predictor of poorer immediate complete or partial response and index lesion specific progression free survival in patients with HCC undergoing TACE or RE. The discrepancy with our results may be due to different locoregional therapies (TACE+RE in their study vs. RE only in our study). On the other hand, Vouche et al. [44], however, observed no significant change in ADC at baseline, 1 and 3 months in patients with HCC post RE ± sorafenib. Furthermore, ADC was not able to predict complete pathological necrosis in their MRI-pathological study including 15 patients.

Recent studies demonstrated ADC changes in the index tumor volume rather than in a single axial plane [19, 24]. A survival benefit was shown in cholangiocarcinoma and neuroendocrine liver metastases treated by TACE exhibiting response by vADC [19, 23, 24]. Bonekamp et al. [19] reported that volumetric functional (vADC and volumetric enhancement) response 3-4w after TACE in patients with HCC showed improved overall survival and was superior to current imaging response criteria (RECIST, mRECIST, and EASL) and AFP [19]. Chapiro et al. [22] found that vADC alone correlated strongly with tumor necrosis at pathologic examination in patients with HCC post TACE.

There is limited data on the role of vADC in HCC post RE, with only one study from Vouche et al. [25]. The authors showed that vADC (mean and standard deviation) significantly increased 4w post RE in 21 patients with HCC and performed better than RECIST in detecting image response post RE using liver explant as reference. However, vADC was not able to predict complete pathological necrosis. They reported vADC (mm^2^/s × 10^− 3^) mean and standard deviation (SD) values of 0.185 and 0.041 at baseline and 1.91 and 0.201 at 4 weeks. In line with their results we observed a significant increase in vADC 6w post RE, however our vADC mean and SD values were higher with 1.288 and 0.325 mm^2^/s × 10^− 3^ at baseline and 1.534 and 0.423 mm^2^/s × 10^− 3^ at 6w. An explanation might be related to differences in b-values used for DWI acquisition (0, 500/50, 500/50, 500, 1000 and 50, 500, 800 in their study vs. 50, 400 and 800 s/mm^2^ in our study).

Most prior studies have reported mean ADC values from a single slice, which do not account for the underlying tumor heterogeneity. Histogram analysis (i.e. first order radiomics) is a new approach for quantifying tumor heterogeneity using routine MRI data. Radiomics, which is defined as the conversion of images to higher-dimensional data and the subsequent mining of these data for improved decision support, appears to offer a nearly limitless supply of imaging biomarkers that could potentially aid cancer detection, diagnosis, assessment of prognosis, prediction of response to treatment, and monitoring of disease status [47]. Histogram analysis refers to a mathematical approach to evaluate gray-level intensity variations within a VOI and may be used to assess intralesional heterogeneity [33]. Studies on different tumors showed that MR histogram analysis could be helpful for diagnosis, biologic aggressiveness evaluation, and therapy response prediction [28–32]. Hu et al. [26] correlated vADC parameters with Ki-67 labeling index in patients with HCC. They found that vADC mean, median 5th, 25th and 75th percentiles demonstrated significant inverse correlations with Ki-67 labeling index. Moriya et al. [27] correlated vADC parameters with histologic grade of HCC. They reported that vADC min showed significant differences among tumor histological grades. ADC min of poorly differentiated HCC was significantly lower than that of combined well and moderately differentiated HCC.

The assessment of treatment response with imaging techniques plays a critical role in the management of HCC. Tumor response criteria may be used as a surrogate marker of efficacy in clinical trials in HCC, and as predictors of survival following RE. It has been suggested that DWI is an earlier surrogate of response compared to size criteria in HCC treated by RE [15–17]. vADC measurements, as early as 3–4 weeks post transarterial chemoembolization, have shown promising results in predicting survival [19] and treatment response [20, 22] in patients with HCC post TACE. Our results suggest that changes in vADC histogram measurements at 6w post RE constitute potential early biomarkers of subsequent treatment response. Future work should include a larger sample size, and should evaluate second order radiomics texture features on DWI and other sequences such as dynamic T1-weighted imaging. Furthermore, inter-platform variability of radiomics features should be assessed.

Our study had several limitations. First, this was a retrospective study. Second, our study population was slightly underpowered showing an overall power of 75% for presence of type II error (false negative rate), reflecting our preliminary experience. Third, the qualitative image analysis was performed by two readers in consensus, which precluded the evaluation of interobserver variability. Interobserver reproducibility for mRECIST was shown to be excellent (ICC of 0.77–0.84) in a recent publication [14]. Fourth, we used enhancement change as surrogate endpoint and did not have histopathological proof of tumor necrosis or data on patient survival. Fifth, we used only one index lesion to classify the response, even though some patients had a large disease burden that may have required response assessment of the entire intrahepatic tumor volume. Sixth, we used different MRI systems and field strengths in our study. However, acceptable inter-platform reproducibility in ADC values has been reported by us and other groups [37–41].

## Conclusion

Our preliminary results indicate that volumetric ADC measurements obtained at 6 weeks and early changes in ADC from baseline are predictive of subsequent response in HCCs treated with RE, while pre-treatment vADC did not have any predictive value.

## Data Availability

The datasets analyzed during the current study are available from the corresponding author on reasonable request.

## Abbreviations

ADC: Apparent diffusion coefficient
CR: Complete response
DWI: Diffusion-weighted imaging
HCC: Hepatocellular carcinoma
mRECIST: Modified response evaluation criteria in solid tumors
PD: Progressive disease
PR: Partial response
RE: ^90^ Yttrium radioembolization
SD: Stable disease
vADC: Volumetric apparent diffusion coefficient
